# The endangered Spitsbergen bowhead whales' secrets revealed after hundreds of years in hiding

**DOI:** 10.1098/rsbl.2020.0148

**Published:** 2020-06-10

**Authors:** Kit M. Kovacs, Christian Lydersen, Jade Vacquiè-Garcia, Olga Shpak, Dmitry Glazov, Mads Peter Heide-Jørgensen

**Affiliations:** 1 Norwegian Polar Institute, Fram Centre, N-9296 Tromsø, Norway; 2 A.N. Severtsov Institute of Ecology and Evolution, Russian Academy of Sciences, Moscow 119071, Russia; 3 Greenland Institute of Natural Resources, DK-1401 Copenhagen K, Denmark; 4 Greenland Institute of Natural Resources, DK-3900 Nuuk, Greenland

**Keywords:** climate changes, conservation, distribution, sea ice, ocean temperatures

## Abstract

Spitsbergen's bowhead whales (*Balaena mysticetus*) were hunted to near extinction in the world's first commercial whaling enterprise; this population clearly remains threatened, but nothing is known about its distribution, making assessment unfeasible. In this study, we document range, movement patterns and habitat preferences of this population, based on tagging done from an icebreaker-based helicopter. Despite their reduced abundance, Spitsbergen's bowhead whales occupy much of their historical range, stretching across the northern Barents Region from East Greenland eastward to Franz Josef Land. Unlike larger bowhead populations to the west, they do not migrate in a classical sense, but rather disperse from wintering grounds in the northernmost parts of their range during spring, returning northward again in autumn, a pattern opposite in terms of directionality compared to other Arctic bowhead whale populations. The extreme affiliation of this population with cold, ice-filled waters is a concern given ongoing climate warming and concomitant rapid sea ice habitat loss.

## 1. Introduction

The Spitsbergen stock of bowhead whales(*Balaena mysticetus*) were hunted to the brink of extinction during the first commercial whaling enterprise, which started in the early 1600s in the Svalbard Archipelago. In the early 1990s, this population was estimated to number in the few tens [1] and its classification remains Endangered on the IUCN Red List today [2]. However, acoustic monitoring in the Fram Strait, between Svalbard and Greenland, demonstrated a year-round presence of bowhead whales in the region, with elaborate and abundant singing taking place 24 h per day in the winter months, suggesting that this drift-ice area in the midst of the southward flowing Arctic Water of the East Greenland Current is a mating ground for this population [3,4]. However, ship-based surveys and a marine mammal sightings database for Svalbard have reported only a few bowheads [5,6]. The track from a single whale tagged in Fram Strait during a bowhead expedition in 2010 reinforced the suggestions by early whalers regarding a very unusual movement pattern in this population: this whale wintered deep in the ice west of Svalbard and went south in the summer, which is opposite to the normal––north in summer and south in winter––seasonal patterns of baleen whales, including bowhead whales, in the Arctic [7]. In late summer 2015, we conducted an aerial survey into the polar ice north of Svalbard using helicopters based on ships, to estimate bowhead whale numbers. Fifteen sightings, involving 27 bowhead whales in the study area, suggested that the Spitsbergen bowhead whale population likely numbers in the low hundreds [8]. This survey further reinforced the strong affiliation this population has with sea ice. The relatively high densities observed suggested the possibility of working more intensively with individuals in this population within their sea ice habitat. Thus, this study was designed to tag bowheads in the Spitsbergen population to determine year-round movement patterns and fine-scale habitat use to address conservation needs and to design future surveys to determine abundance across the range of this endangered population.

## 2. Material and methods

Because Spitsbergen bowhead whales are highly ice affiliated, the novel use of a helicopter tagging platform (based on a ship) was the only logistically functional option. We used an Écureuil Eurocoper AS350 to search for and approach the whales in the drifting sea ice of the western Fram Strait in late May–early June 2017. Sixteen Spot 5 satellite transmitters (https://wildlifecomputers.com/) were deployed using an ARTS (Aerial Rocket Transmitter System) [9] air gun (12–14 bar pressure) from a distance of approximately 10 m. The custom-designed darts were 30 cm in length, and thus penetrated only the skin and blubber layer (i.e. did not reach the muscle). Generalized additive mixed effect models (GAMMs) were used to study movement and space use (via calculating time spent in area (TSA)) relative to environmental conditions within seasons. Details regarding the sampling regime, data filtering, extraction and calculation of environmental variables and statistical analyses can be found in the electronic supplementary material.

## 3. Results

**Figure 1.**
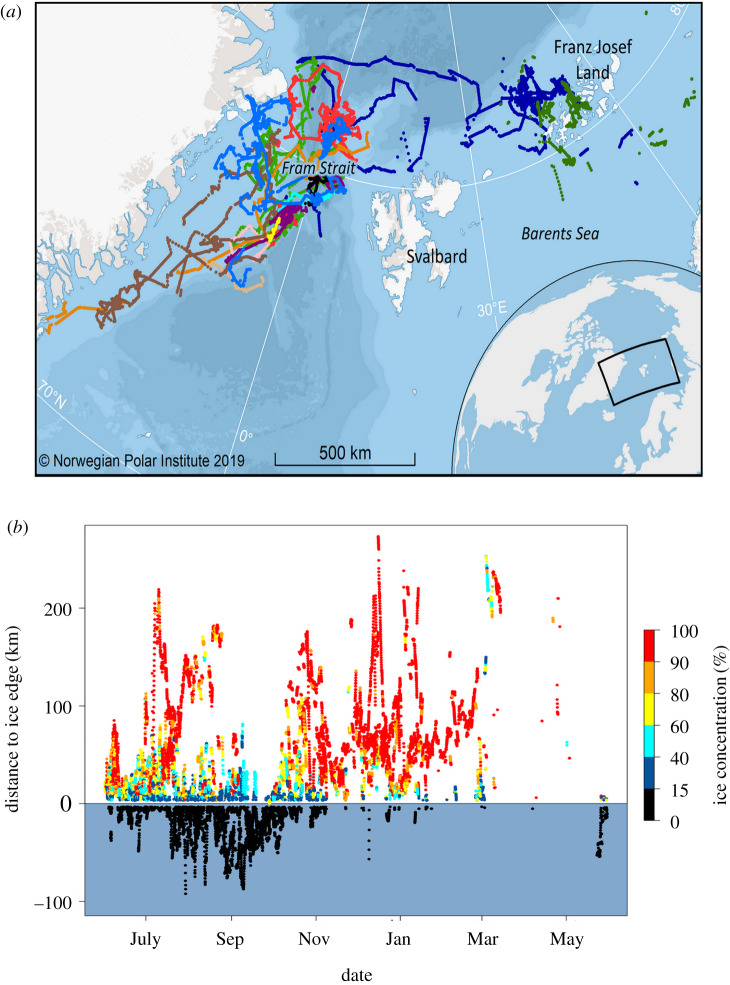
(*a*) Interpolated hourly positions along track segments (1 June 2017–31 May 2018) from 13 bowhead whales tagged in Fram Strait. (*b*) Distance to the ice edge versus date for 13 bowhead whales tagged in Fram Strait. The white background in the top of the figure indicates that the whales are inside the ice edge, whereas a blue background at the bottom of the figure indicates locations in open water. Locations are colour coded according to sea ice concentration.

**Figure 2.**
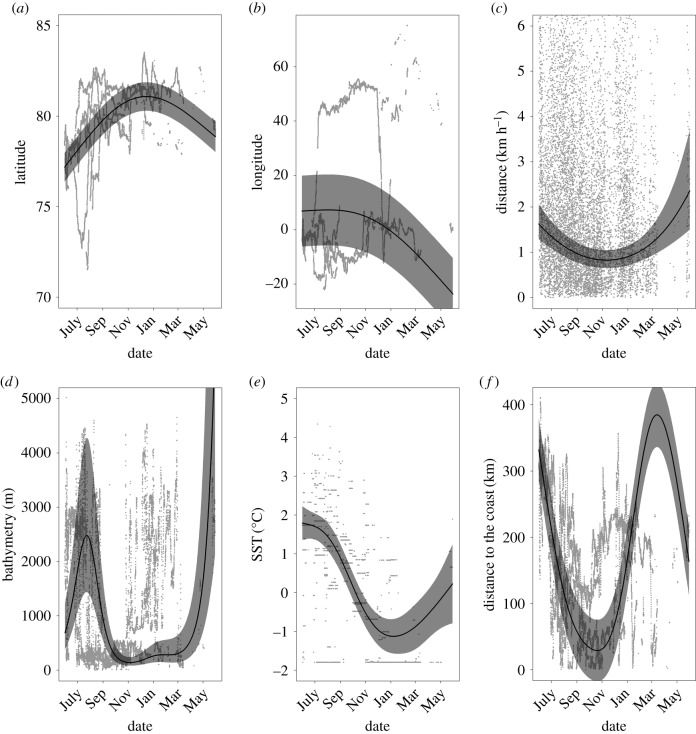
GAMM model outputs comparing (*a*) latitude, (*b*) longitude and (*c*) distance travelled/hour, (*d*) bathymetry (*e*) sea surface temperature and (*f*) distance to the coast versus date for 13 bowhead whales tagged in Fram Strait. Fitted estimates from models (solid curves) are represented along with the 95% CIs (polygons).

Thirteen of the tagged bowhead whales provided location information; mean record length was 181 ± 199 days (range 4–709 days). Following our application of a speed, distance, angle filter and selection of a one-year study period (see electronic supplementary material, table S1), we retained 12 201 locations (electronic supplementary material, figure S1). Subsequent to creating 1 h interpolations within the identified track segments (defined as sections of tracks where there were no gaps in transmission greater than 24 h; see electronic supplementary material, figure S2), 22 489 location estimates were obtained, 22 268 of which were retained for habitat assessment because they could be associated with oceanographic parameters (figure 1*a*). During the study period, the animals were exposed to highly variable environmental conditions ranging from shallow, coastal areas to deep (max. 5018 m) offshore (max. 411 km) areas. The whales traversed areas with sea surface temperatures ranging from −1.8°C to 4.34°C (mean = 0.29 ± 1.52°C), with sea ice concentrations ranging from open water to 100% coverage. We tagged all of the animals in a restricted area in central Fram Strait (close to 78°N, 0°), but they did not migrate directionally in any classical sense; they simply dispersed north and south, east and west (figures 1*a* and 2*a,b*), at relatively high swimming speeds (1–2 km h^−1^) over the summer period (figure 2*c*). Eleven of the whales stayed west of Svalbard, while the other two moved eastward to Franz Josef Land and somewhat beyond (figure 1*a* and electronic supplementary material, figure S3). During the autumn, movements were more homogeneously northward, and speed of travel was reduced (figure 2*a,c* and electronic supplementary material, figure S4). All of the whales spent the winter in relatively small areas in waters off Northeast Greenland or Franz Josef Land (electronic supplementary material, figure S4). Toward the end of winter and during the spring, the whales started moving more quickly once again, generally in a southward direction (figure 2*a,c*).

We explored movement patterns and habitat use throughout the year using GAMMs (details regarding model selection, model diagnostics and model outputs can be found in the electronic supplementary material, tables S2, S3, figures S5 and S6). The GAMMs showed that during summer, the whales occupied the marginal ice zone (MIZ, the edge of which is defined as the extent of at least 15% ice cover) in deep, offshore areas over the continental slope, where relatively high ice concentrations prevailed, with sea surface temperatures (SST) averaging 2°C (figures 1*b* and 2*d–f*; electronic supplementary material figure S7). In autumn, the whales moved into shallower areas, closer to coastlines and spent more time in lighter ice concentrations or open water areas, but SSTs were progressively colder, reaching below zero by October (figures 1*b* and 2*e–f*; electronic supplementary material, figure S7). As winter approached the animals returned to deeper, offshore areas where they occupied areas with high sea ice concentrations, often exceeding 90%, with SSTs below zero (figures 1*b* and 2*d–f*; electronic supplementary material, figure S7). In spring, few tags were still providing locations, but the whales for which we have data occupied areas with progressively declining ice concentrations and progressively higher SST values (temperatures became positive), although they remained offshore in deep, relatively cold areas (figure 2*e* and electronic supplementary material, figure S7). In all seasons, the whales spent most of their time inside the margins of the drifting ice, though during late summer and early autumn they made forays into open water (figure 1*b*).

GAMMs investigating TSA relative to environmental conditions within seasons suggested that depth was important in summer, autumn and winter, but the whales changed preference according to season, particularly spending more time in shallow areas in the autumn (electronic supplementary material, figure S8; also see electronic supplementary material figure S4). Proximity to coastlines also appeared to be an important variable influencing TSA; the whales were closest to the coast in autumn and furthest from the coast in winter (electronic supplementary material, figure S8). TSA was impacted by distance to the ice edge, particularly in the winter when the whales spent most of their time deep inside the ice, far from the edges (electronic supplementary material, figure S8). SST was also an important variable with regard to TSA; the whales spent most of their time in cold water, even during summer (SST < –1°C), and maintained this general cold water preference through autumn and winter, when they stayed longer periods of time in water with SSTs between −1°C and +1°C (electronic supplementary material, figure S8). TSA models' output summaries can be found in electronic supplementary material, table S4.

## 4. Discussion

In all seasons, the bowheads spent most of their time in cold water and in close association with sea ice. This species is the only baleen whale that resides year-round in the Arctic, and through evolutionary time they have become the most specialized baleen whale species. Not surprisingly, most of their unique characteristics are associated with their high latitude, cold water, ice-affiliated lifestyle [10]. This includes having no dorsal fin and having a slow, conservative life-history strategy that involves extreme longevity (up to 200 yr), late sexual maturation (20 yr) and a long inter-calf interval (4–7 yr). Additionally, their 4 m long baleen permits ingestion rates during summer and fall that allow for maintenance of a blubber layer that can be up to 50 cm thick. The need to feed heavily on lipid-rich Arctic copepods, euphausiids, amphipods and mysids during the Arctic summer is thought to be the main reason for the northward migration that most bowhead whale populations undertake. Bowhead whales in the Bering–Chukchi–Beaufort and in the Eastern Canada–West Greenland populations move northward in summer along predictable migration corridors [11,12]. Only the Spitsbergen bowhead whale population is known to spend the winter at its northern-most latitudes, moving southward in summer. Although this population occupies the MIZ in the summer and autumn, presumably to take advantage of the production produced by upwelling and ice-melt related phenomena, it does not get pushed south with the ice edge in winter. Instead, individuals move north and remain up to hundreds of kilometres inside the ice edge, in areas classified as having 90–100% ice concentrations. Coastal polynyas in Franz Josef Land in the eastern part of the Spitsbergen bowhead's range and along the Northeast coast of Greenland (e.g. North East Water Polynya) and flaw lead systems maintained by the powerful, southward flowing East Greenland Current are likely important determinants of habitat suitability in winter. Occasional incursions of North Atlantic Water in the Fram Strait likely also play a role in keeping enough cracks and leads open in northerly waters of this region, such that they can be winter habitat for bowheads.

The history of human exploitation of this population might play a part in the habitat preference we see in Spitsbergen's bowhead whales in modern times. Coastal and pelagically inclined bowhead whales in the Spitsbergen population were likely extirpated, leaving only individuals that tended to reside in ice protected refugia [13]. Mammal populations that have been reduced to tiny fractions of their former population sizes usually occupy only an edge of their former range [14]. However, we show in this study that this is not the case for Spitsbergen bowhead whales. These whales spread across most of the northern Barents Sea despite their low population numbers, although they remain rare in the Svalbard Archipelago, which was core habitat in the past [5,6]. The minimal cost of transport in the marine environment and the bowhead whale's ability to communicate across distances of many tens of kilometres, potentially up to hundreds of kilometres [15], might facilitate the broad geographic spread we see in this small population.

Bowhead whales are shallow divers that feed on a variety of Arctic crustaceans, primarily in the top 200 m, although they can dive deeper than 500 m [16]. Although we know nothing specific about the diet or seasonal feeding patterns of Spitsbergen bowheads, it is reasonable to assume that they feed primarily during the daylight period, when zooplankton ascend in the water column and when this and other bowhead populations tend to be in shallower areas up on coastal shelves [17]. However, several recent studies suggest that bowhead whales might feed year-round [18,19] and the fact that some calanoid copepod stages occupy intermediate depths in under-ice environments in winter could facilitate winter feeding [20].

Both the Bering–Chukchi–Beaufort and the Eastern Canada West Greenland bowhead populations are recovering from historical overexploitation, and there are a growing number of promising signs for the Spitsbergen population [8,13,21–23], though data is not yet available over time frames meaningful for trend assessment in the latter area. Decreased sea ice owing to global warming will likely promote greater mixing between bowhead whale populations across areas previously covered by continuous ice [24], as this sort of connectivity in the past has been suggested by genetics studies [25]. Narrow ranges of preferred, cold SSTs have been noted for other bowhead whale populations [26,27] and seem to be the case for Spitsbergen bowhead whales according to our SST and TSA analyses. This creates a serious concern that ongoing increases in water temperatures in the Greenland and Barents Seas and concomitant sea ice losses [28,29] might become critical in terms of habitat loss and thermal stress. Reduced ice cover also creates the potential for increased mortality owing to killer whale predation, which is already a serious issue for the Sea of Okhotsk population [30]. Disease exposure, food web changes that result in less food availability (especially fat-rich calanoid copepods) and increased human traffic in the Arctic are also concerns [31]. If Spitsbergen bowhead whales retain their strong preference for cold, ice-covered waters, which we have demonstrated herein, their distribution will retract north to offshore, deep water areas in the future, where they would be forced to rely on pelagic zooplankton production; such a situation is likely to prevent recovery of the population.

## Supplementary Material

Methodological details and supplementary figures and Table

## Data accessibility

Data are available at the National Polar Data Centre for Norway https://doi.org/10.21334/npolar.2020.395011fc.
