# Supplementary material for: The endangered Spitsbergen bowhead whales' secrets revealed after hundreds of years in hiding
Source: Biol Lett. 2020 Jun 10;16(6):20200148. doi: 10.1098/rsbl.2020.0148 (PMC7336847; doi:10.1098/rsbl.2020.0148)
Supplement: Methodological details and supplementary figures and Table [file rsbl20200148supp1.docx]

Supplementary material

**Methodology**

The satellite transmitters deployed on the bowhead whales were housed in stainless-steel cylinders that were attached to the whales dorsally (approximately half way between the head and the tail) with a four-bladed point attached to a 30 cm long rod, which was held in place in the tissue with 4 sets of barbs and 6 backward-facing petals. Tags were surgically sterilized, and the anchoring system was coated with Gentamicyn sulfate antibiotic prior to implantation. The tags were programmed to start searching for satellites at 06:00 hrs daily, and to try up to 200 times. They then closed operations until the next day. All data processing and analyses were done using the R statistical framework (R 3.6.0). Satellite derived locations were filtered using a speed, distance and angle filter (SDA filter; [1,2] using the R package ‘argosfilter’ (http://cran.r-project.org)). This filter removes all low precision (LC Z) points as well as those requiring unrealistic swimming speeds or unlikely turning angles; the swimming speed threshold was set at 3 m/s and all spikes with angles smaller than 15 or 25 degrees were removed if their lengths were greater than 2.5 or 5 km, respectively [3,4].

Filtered tracks were divided into segments if we did not have a location within a 24 h period, because the bowhead tracks were quite patchy due to their use of ice-filled waters and their ability to breathe through cracks with only their elevated nostrils exposed (and not the tag). A total of 75 segments from the 13 animal’s tracking records were identified in the one year study period selected for analyses (01 June 2017 – 31 May 2018). Filtered locations were subsequently interpolated within segments at 1 h intervals along the trip-line to avoid transmission biases. Locations estimated to occur on land were removed using the 1:10 m - file.

Longitude (LON) and latitude (LAT) of retained interpolated locations, as well as respective distance between them within each segment (DIST, km), were extracted. Each filtered, interpolated location was assigned to a season - locations occurring June-August were assigned to Summer, September-November were assigned to Autumn, December-February were assigned to Winter and March-May were assigned to Spring.

Extraction and calculation of the environmental variables

Five environmental variables, bathymetry (DEP, m), sea ice concentration (ICE, %), distance to the ice edge (ICE EDGE, km), sea surface temperature (SST, °C), and distance to the nearest coast (COAST, km), were calculated for each interpolated location based on their locations and time-stamps. DEP was extracted from the 500 m grid resolution International Bathymetric Chart of the Arctic Ocean Version 3.0 (IBCAO; [5]. ICE was extracted from the daily 6.25 km grid resolution AMSR-E ice remote sensing system [6]. ICE EDGE was calculated from ICE using Qgis (the ice edge was set at 15 % ice concentration). Positive values of ICE EDGE mean that locations were inside the ice while negative values for ICE EDGE reflect open ocean locations (until the 15 % concentration limit). SST was extracted from the monthly 2° grid resolution from the Extended Reconstructed Sea Surface Temperature (ERSST) v5 [7]. Finally, COAST was calculated using the same land file described previously (1:10 m-[www.naturalearthdata.com](http://www.naturalearthdata.com/)). Interpolated locations with unavailable environmental variables were removed.

Time spent in area

The intermittent nature of location transmissions negated the use of classical methods used to quantify space use, such as First Passage Time [8,9] or State Space Models [10-12], so we calculated time spent in area manually (TSA; [13]. Time spent (h) within a 5 x 5 km grid-square was calculated monthly for each individual as the sum of the interpolated hourly locations within each grid cell. Environmental variables previously described (DEP, ICE, ICE EDGE, SST and COAST) were extracted for each cell as the mean of environmental values corresponding to the interpolated locations occurring within the cells for each individual.

Modelling approach

To explore seasonality, the three movement metrics (LON, LAT, DIST) as well as the five environmental metrics (DEP, ICE, ICE EDGE, SST, COAST) were investigated separately in relation to the day of the year (the number of days since the 1^st^ of June (i.e. the first day of the selected one-year period – day hereafter)). Generalized additive mixed models (GAMM; `uGamm' function in the R package `MuMIn' that called ‘gamm’ function in the R package `mgcv) were used to study relationships; day of the year was included as a smooth term (k was set at 3 or 6 for the models involving the movement and environmental variables, respectively). Individual ID was included as both a random effect and as a grouping factor in the temporal autocorrelation structure of the order one (corAR1) term. We used a Gaussian family distribution to fit models; DIST, DEP and ICE were log transformed to attempt to meet model assumptions of homoscedasticity and normality of residuals (log(variable+1) for ICE since 0 values were present). We did model selection according to the Akaike's information criterion, AIC [14]. Diagnostics of the selected models as well as temporal and spatial autocorrelation assessment plots were used to validate final models [14, 15].

To quantify space use relative to environmental conditions within seasons, monthly TSA was modelled using generalized additive mixed models (GAMM; ‘gamm’ function in the R package `mgcv) in relation to DEP, ICE EDGE, SST and COAST as well as Season. We did not include ICE in the models since it was highly correlated with ICE EDGE (Pearson correlation 73%). Positive values of TSA were retained for analysis since a value of 0 in a cell does not necessarily mean that the cell was not used by animals. Environmental variables were included as smooth terms (k was set at 4 for those) while Season was included as a “by-variable” (i.e. environmental variables smooth curves were made for each Season). Before we ran models, explanatory variables were standardized - to have a mean of 0 and a standard deviation of 1 - and models were fitted with a Negative binomial family distribution (theta was fixed at 1.602) since TSA was over-dispersed, count data. Individual IDs were included in the models as both a random effect and as a grouping factor in the temporal autocorrelation structure of the order one (corAR1) term. We conducted model selection and model validation using the confidence intervals of the corresponding smooth curves, as recommended by [14].

**References**

1. Freitas C, Lydersen C, Fedak MA, Kovacs KM. 2008. A simple new algorithm to filter marine mammal Argos locations. *Mar. Mamm. Sci.* **24**, 315–325.

2. Freitas, C. 2012. Argosfilter package. <https://cran.rproject.org/web/packages/argosfilter/argosfilter.pdf>.

3. Quakenbush LT, Citta JJ, George JC, Small RJ, Heide-Jørgensen MP. 2010. Fall and winter movements of bowhead whales (*Balaena mysticetus*) in the Chukchi Sea and within a potential petroleum development area. *Arctic* **63**, 289–307.

4. Heide-Jørgensen MP, Laidre KL, Nielsen NH,Hansen RG, Røstad A. 2013. Winter and spring diving behaviour of bowhead whales relative to prey. *Anim. Biotelem.* **1**, 15.

5. Jakobsson M *et al*. 2012. The International Bathymetric Chart of the Arctic Ocean (IBCAO) Version 3.0, *Geophysical Research Letters*, doi: 10.1029/2012GL052219.

6. Spreen G, Kaleschke L, Heygster G. 2008. Sea ice remote sensing using AMSR-E 89 GHz channels. *J. Geophy. Res*. **113**, C02S03. (doi:10.1029/2005JC003384)

7. Huang B, Thorne PW, Banzon VF, Boyer T, Chepurin G, Lawrimore JH, Menne MJ, Smith TM, Vose RS, Zhang H-M. 2020. Extended Reconstructed Sea Surface Temperature version 5 (ERSSTv5), Upgrades, validations, and intercomparisons. NOAA National Centers for Environmental Information, doi:10.7289/V5T72FNM [access 19 January 2020].

8. Fauchald P, Tveraa T. 2003. Using first-passage time in the analysis of area-restricted search and habitat selection. *Ecology* **84**, 282-288. (doi:10.1175/JCLI-D-16-0836.1)

9. Fauchald P, Tveraa T. 2006. Hierarchical patch dynamics and animal movement pattern. *Oecologia* **56**, 234–238. (doi:10.1007/s00442-006-0463-7)

10. Jonsen ID, Mills Flemming J, Myers RA. 2005. Robust state-space modelling of animal movement data. *Ecology* **86**, 2874–2880.

11. Patterson TA, Thomas L, Wilcox C, Ovaskaien O, Mattiopoulos J. 2008. State–space models of individual animal movement. *TREE* **23**, 87-94. (doi:10.1016/j.tree.2007.10.009)

12. Schick RS, Loarie SR, Colchero F, Best BD, Boustany A, Conde DA, Halpin PN, Joppa LN, McClellan CM, Clark JS. 2008. Understanding movement data and movement processes: current and emerging directions. *Ecol. Lett.* **11**, 1338-50. (doi:1111/j.1461-0248.2008.01249.x)

13. Sumner MD, Wotherspoon SJ, Hindell MA. 2009. Bayesian Estimation of Animal Movement from Archival and Satellite Tags. *PLoS ONE* **4**, e7324. (doi:10.1371/journal.pone.0007324)

14. Zuur AF, Ieno EN, Walker NJ, Saveliev AA, Smith GM. 2009. Mixed Effects Models and Extensions in Ecology with R. New York, NY: Springer-Verlag.

15. Zuur AF, Ieno EN, Elphick CS. 2010. A protocol for data exploration to avoid common statistical problems. *Methods Ecol. Evol.* **1**, 3−14 (doi: 10.1111/j.2041-210X.2009.00001.x)


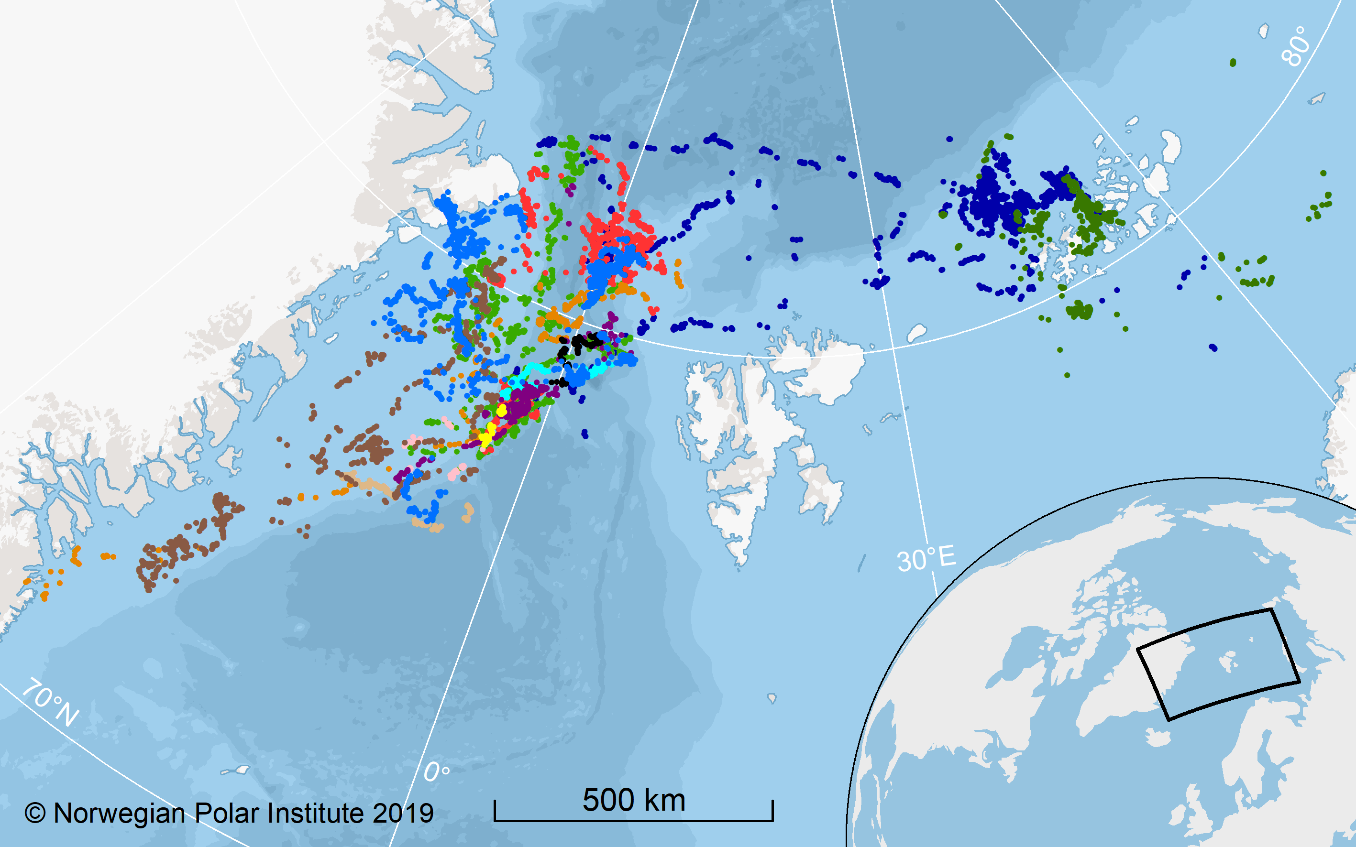


Figure S1. SDA filtered locations (01 June 2017 – 31 May 2018) for 13 bowhead whales tagged in Fram Strait in June 2017. Colors indicate individual animals.


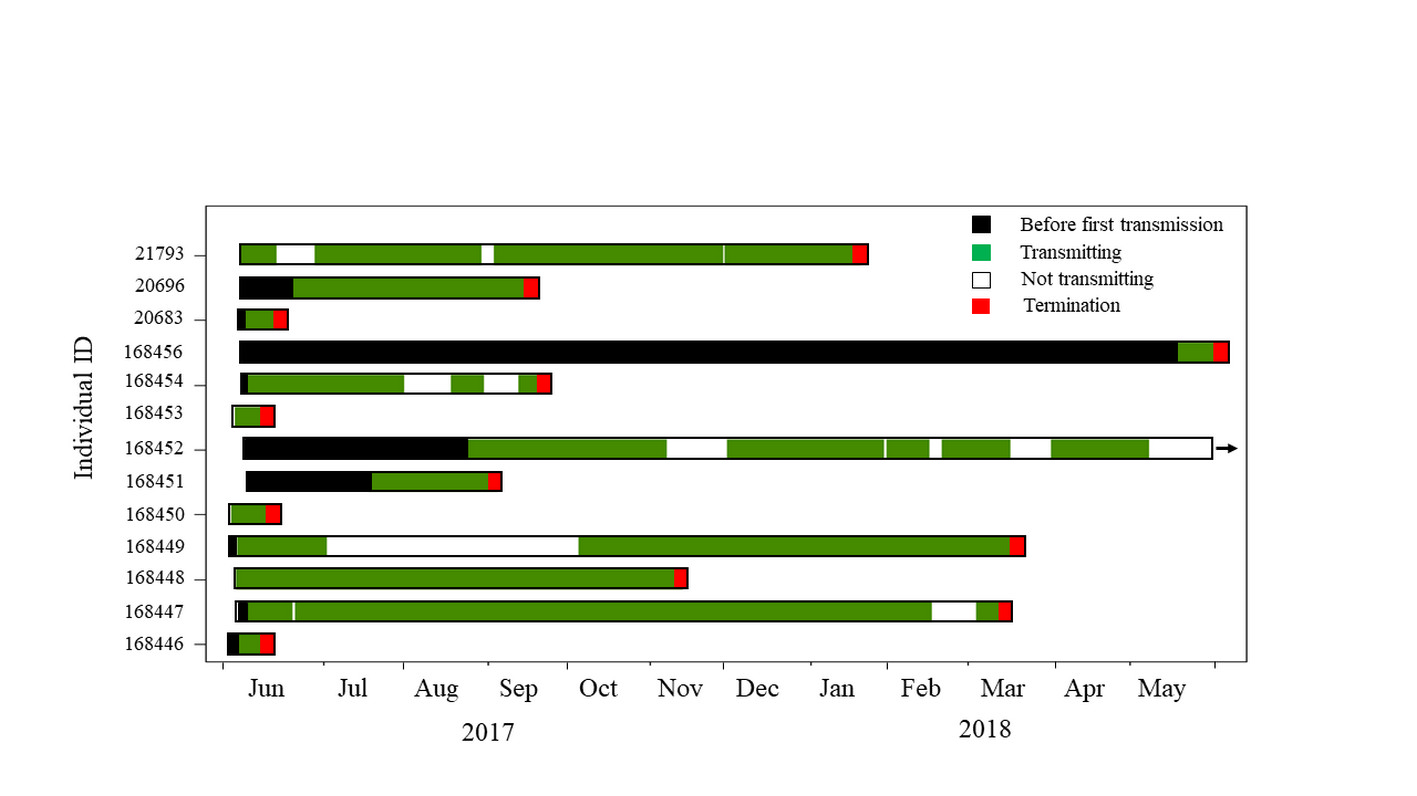


Figure S2. Location transmission records for 13 bowhead whales tagged in Fram Strait from in late May and early June 2017 – for the period 01 June 2017 until 31 May 2018. (The complete record for animal no. 168452, which extended into May 2019, is depicted in detail in Fig. S3).


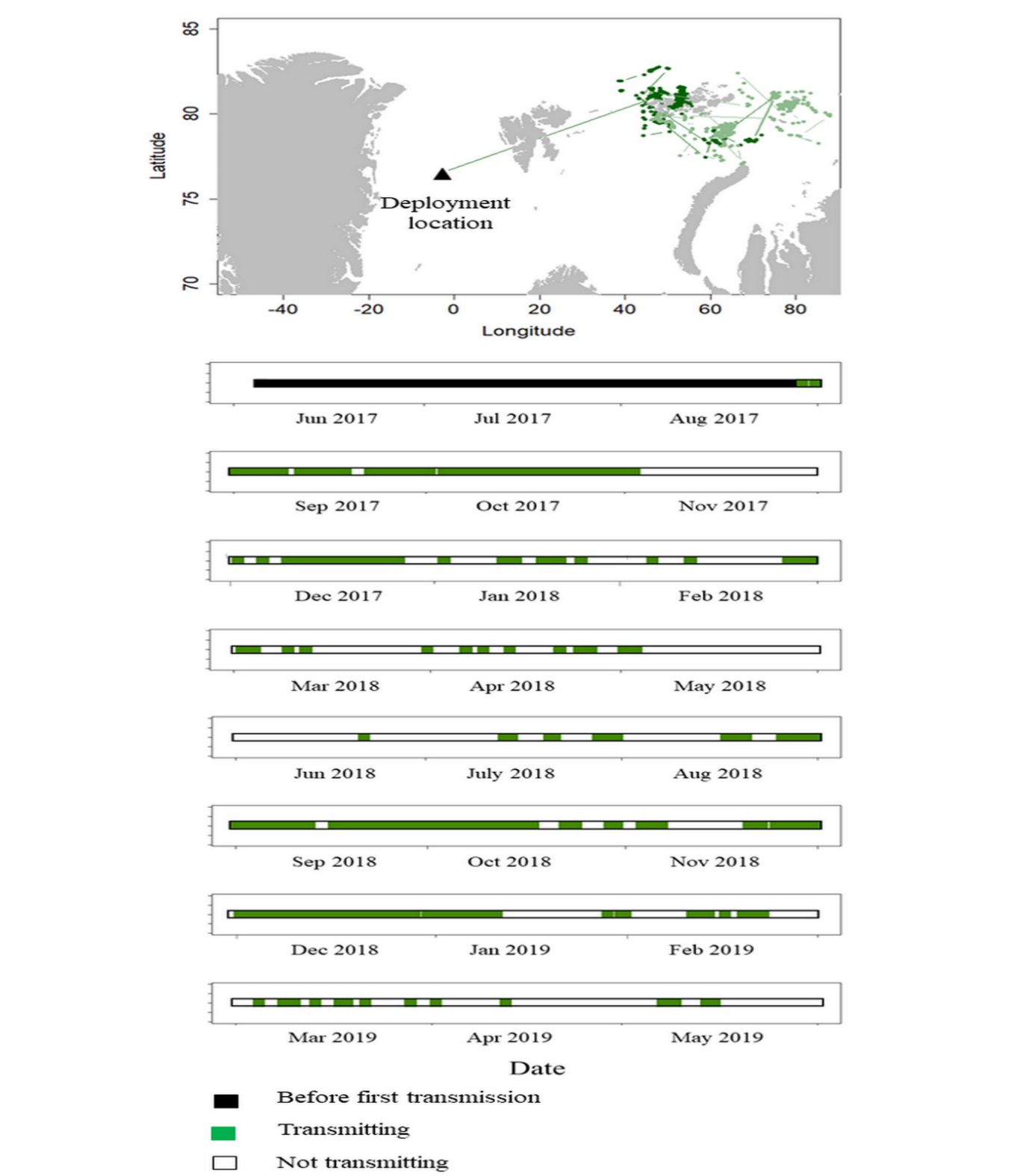


Figure S3. Entire track for bowhead whale no ID-168452, tagged in Fram Strait in June 2017. The tag sent the first location in August 2017 at which time the whale had moved to Franz Josef Land in Russia. The triangle on the map indicates where this whale was tagged. The dark green dots are locations used in this study and the light green dots go beyond the study period (until May 2019). The bar chart under the map depicts - the period between tagging and the first location (black), days with transmission of locations (green) and gaps in the record of more than 24 hours (white).


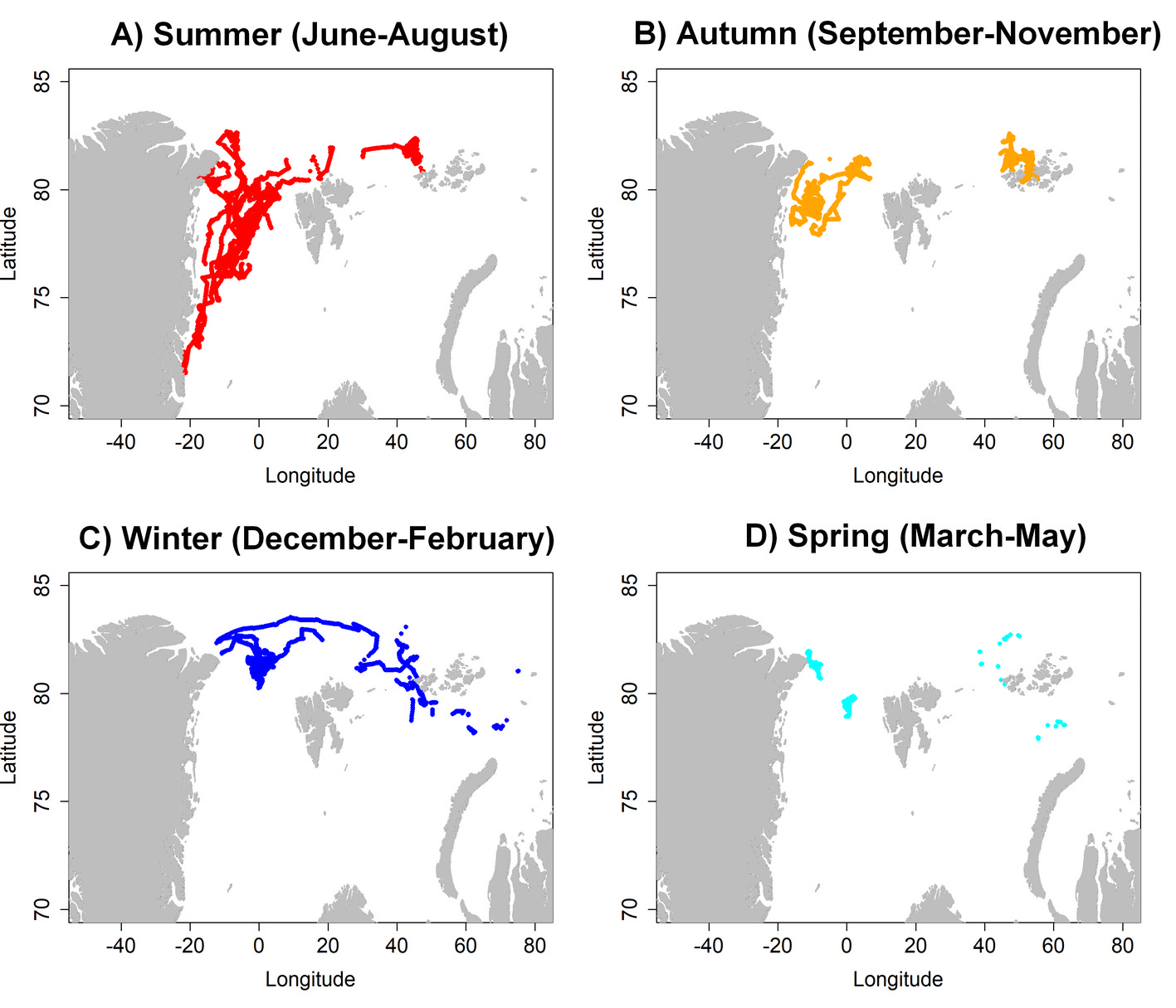


Figure S4. Latitude-longitude of positions interpolated hourly along track segments for 13 bowhead whales tagged in Fram Strait plotted according to season: A) summer; B) autumn; C) winter and; D) spring.


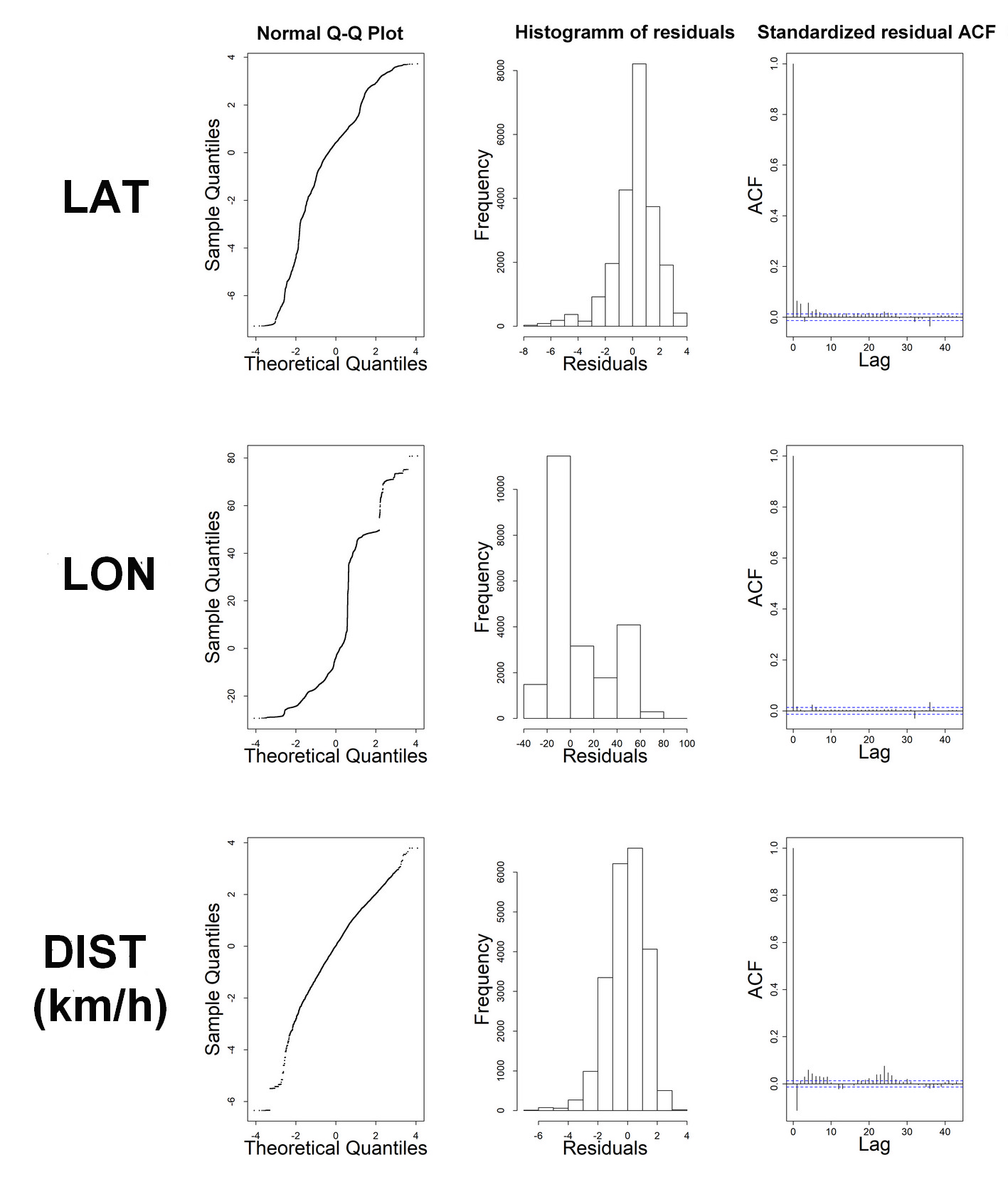


**Figure S5.** Diagnostic residual plots (Normal Q-Q Plot, Histogram of residuals and Standardized residual ACF) for the selected models investigating movement metrics relative to date.


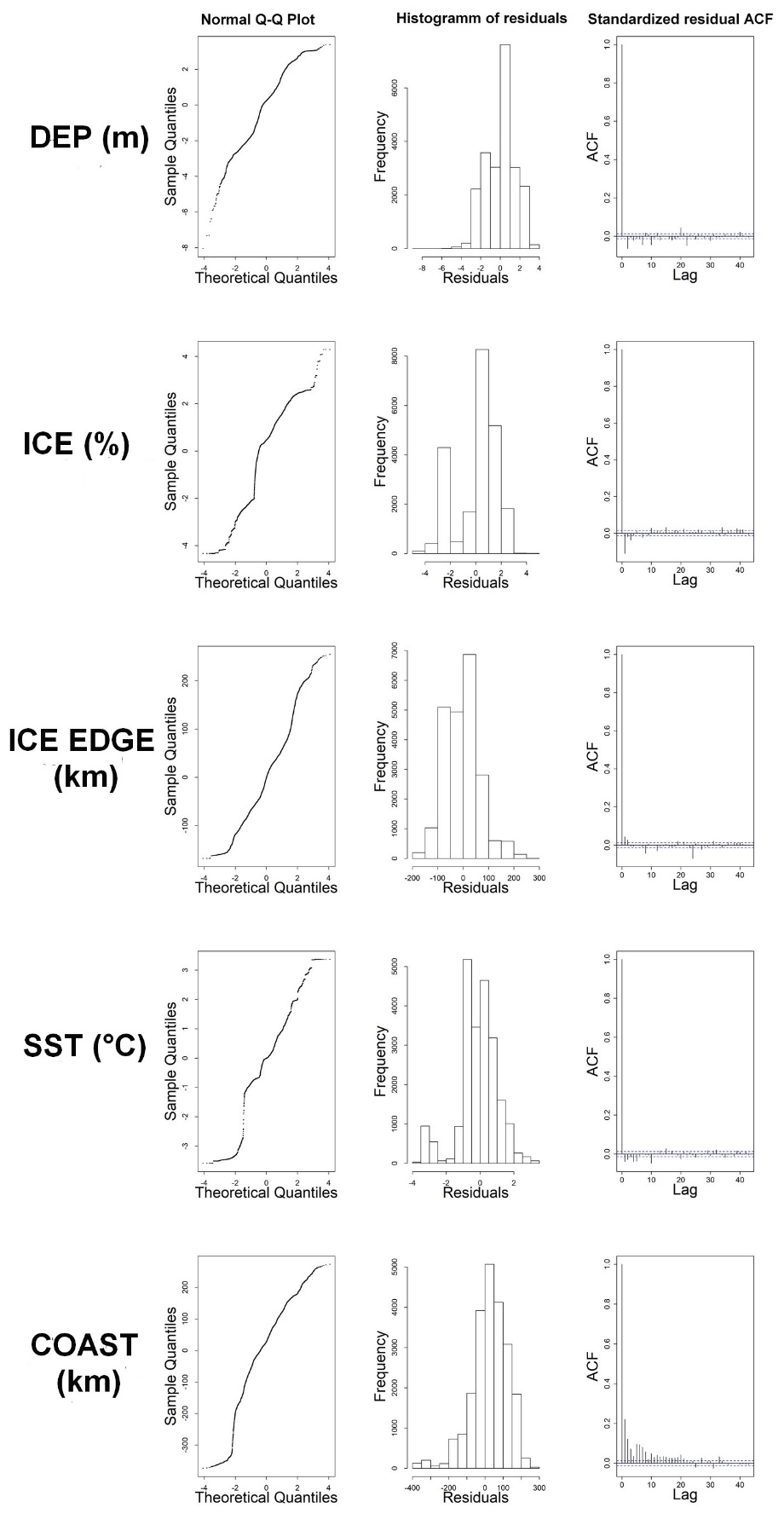


**Figure S6.** Diagnostic residual plots (Normal Q-Q Plot, Histogram of residuals and Standardized residual ACF) for the selected models that investigate environmental metrics relative to date.


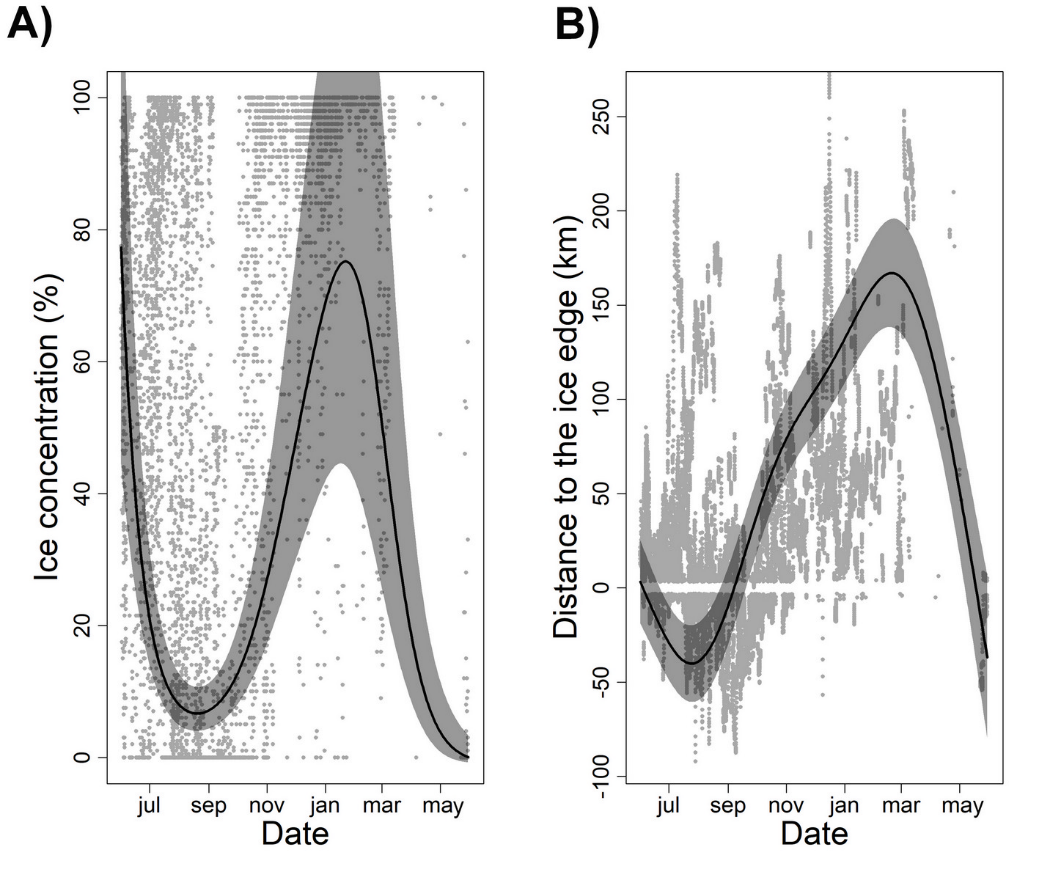


**Figure S7**. GAMM model outputs comparing A) sea ice concentration and B) distance to the ice edge vs date for 13 bowhead whales tagged in Fram Strait. Fitted estimates from models (solid black curves) are represented along with the 95 % CIs (dark grey polygons). Positive values of distance to the ice edge mean that locations were inside the ice while negative values for ice edge reflect open ocean locations (less than 15% sea ice concentrations)


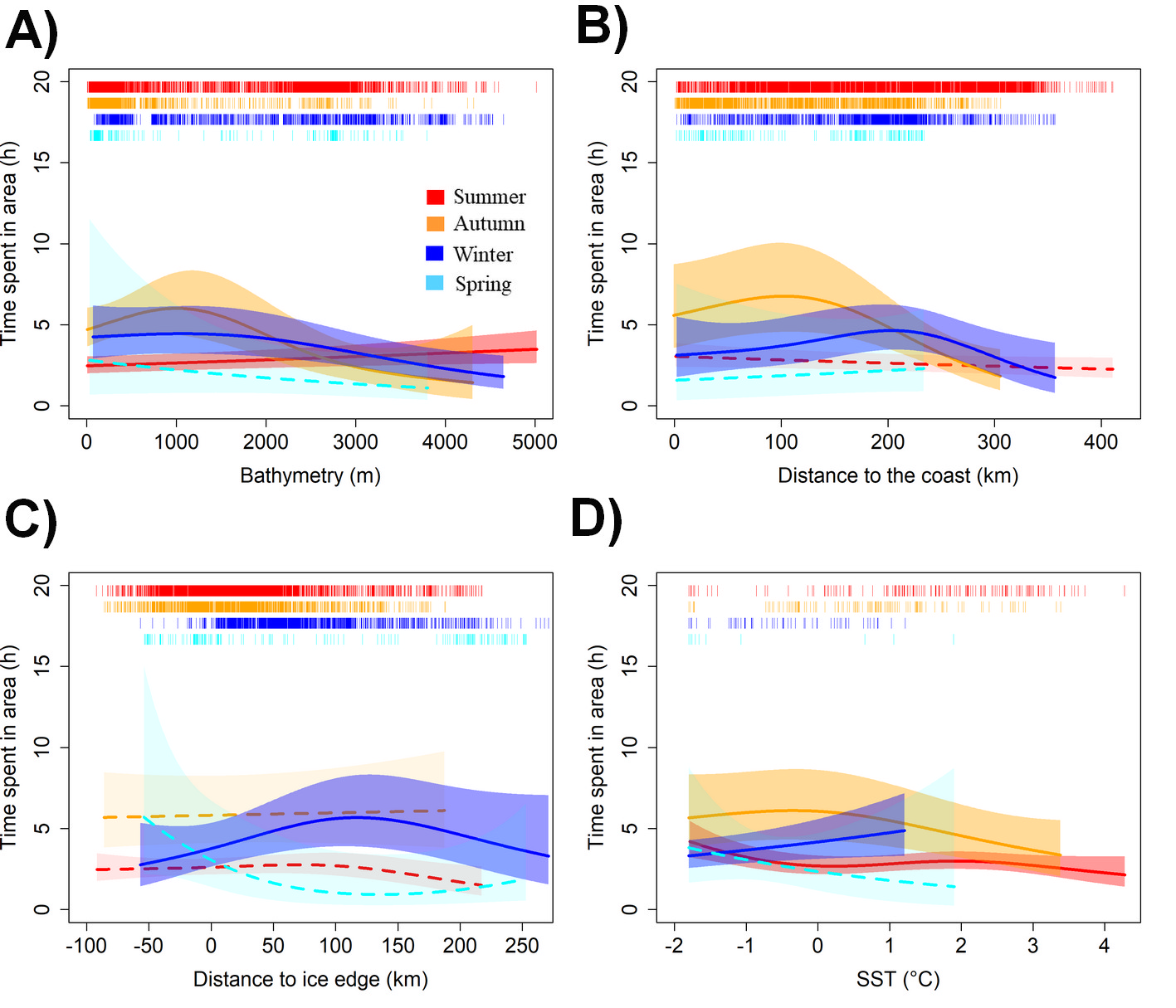


**Figure S8**. GAMM model outputs comparing TSA for 13 bowhead whales tagging in Fram Strait with respect to A) bathymetry, B) distance to the coast, C) distance to the ice edge, and D) SST. Fitted estimates from model are represented along with the 95 % CIs (polygons). Statistically significant relationships are depicted with solid lines, whereas dotted lines are used for relationships not meeting p≤0.05. Positive values of distance to the ice edge mean that locations were inside the ice while negative values for ice edge reflect open ocean locations (less than 15 % sea ice concentration). The seasonally colour codes lines at the top of each illustration show the distribution of available data.

**Table S1.** Details regarding tag deployments and data records (after track filtration and selection of one complete year) for 13 bowhead whales tagged in Fram Strait including ID number, deployment date, deployment location (longitude, latitude), date of first transmission, total duration of data record, number of segments (parts of the total record of individuals without gaps of more than 24 hr), mean segment duration and SD for segment durations.

| ID | Deployment date (d/m/y) | Longitude (west of O^o^) | | Latitude  (^o^N) | | Date of first transmission  (d/m/y) | Duration  (d) | | Number of segments | Mean segment duration (d) | SD segment  duration  (d) |
| --- | --- | --- | --- | --- | --- | --- | --- | --- | --- | --- | --- |
| 168446 | 01/06/2017 | | 4.37 | | 77.32 | 03/06/2017 | 2 | | 1 | 2 | NA |
| 168447 | 03/06/2017 | | 3.91 | | 76.88 | 07/06/2017 | | 276 | 11 | 19 | 41 |
| 168448 | 03/06/2017 | | 3.82 | | 76.90 | 03/06/2017 | 158 | | 5 | 30 | 60 |
| 168449 | 01/06/2017 | | 4.38 | | 77.32 | 03/06/2017 | 284 | | 16 | 10 | 11 |
| 168450 | 30/05/2017 | | 4.73 | | 77.97 | 30/05/2017 | 7 | | 1 | 7 | NA |
| 168451 | 04/06/2017 | | 3.55 | | 76.57 | 04/06/2017 | 38 | | 2 | 17 | 16 |
| 168452 | 05/06/2017 | | 3.09 | | 76.53 | 29/08/2017 | 247 | | 30 | 3 | 6 |
| 168453 | 02/06/2017 | | 4.68 | | 77.14 | 02/06/2017 | 4 | | 1 | 4 | NA |
| 168454 | 04/06/2017 | | 4.25 | | 76.68 | 06/06/2017 | 103 | | 6 | 8 | 13 |
| 168456 | 04/06/2017 | | 4.07 | | 76.06 | 23/05/2018 | 7 | | 1 | 7 | NA |
| 20683 | 04/06/2017 | | 3.06 | | 76.59 | 05/06/2017 | 6 | | 1 | 6 | NA |
| 20696 | 04/06/2017 | | 4.07 | | 76.06 | 23/06/2017 | 82 | | 3 | 27 | 20 |
| 21793 | 04/06/2017 | | 4.25 | | 76.69 | 04/06/2017 | 224 | | 10 | 15 | 20 |

**Table S2.** Degrees of freedom (df) and AIC values for models investigating movement (LAT, LON, DIST) and environmental (DEP, ICE, ICE EDGE, SST, COAST) metrics relative to date (day).

| **Movement metrics** | | | |
| --- | --- | --- | --- |
| ***LAT*** | ***s(day)*** | ***df*** | ***AIC*** |
|  | + | 6 | -71672.9 |
|  |  | 4 | -68005.1 |
| ***LON*** | ***s(day)*** | ***df*** | ***AIC*** |
|  | + | 6 | 9219.9 |
|  |  | 4 | 9928.4 |
| ***DIST*** | ***s(day)*** | ***df*** | ***AIC*** |
|  | + | 6 | 48526.7 |
|  |  | 4 | 48568.0 |
| **Environmental metrics** | | | |
| ***DEP*** | ***s(day)*** | ***df*** | ***AIC*** |
|  | + | 6 | -25822.2 |
|  |  | 4 | -25376.8 |
| ***ICE*** | ***s(day)*** | ***df*** | ***AIC*** |
|  | + | 6 | 24633.1 |
|  |  | 4 | 24726.8 |
| ***ICE EDGE*** | ***s(day)*** | ***df*** | ***AIC*** |
|  | + | 6 | 140528.1 |
|  |  | 4 | 140967.5 |
| ***SST*** | ***s(day)*** | ***df*** | ***AIC*** |
|  | + | 6 | -10770.5 |
|  |  | 4 | -10564.8 |
| ***COAST*** | ***s(day)*** | ***df*** | ***AIC*** |
|  | + | 6 | 115172.3 |
|  |  | 4 | 121751.4 |

**Table S3.** GAMM model output summaries investigating movement (LAT, LON, DIST) and environmental metrics (DEP, ICE, ICE EDGE, SST, COAST) as a function of the date (day). The estimates, *t-values* and *p-values* are shown for the linear predictor variables and the estimated degrees of freedom (edf), F and *p-values* are shown for the predictor variables included in the smooth function in each model. The level of temporal autocorrelation (Phi value) is also given.

| **Movement metrics** | | | | | | | | |  |
| --- | --- | --- | --- | --- | --- | --- | --- | --- | --- |
|  |  | ***estimate*** | ***t-value*** | ***p-value*** |  | ***edf*** | ***F*** | ***p-value*** | ***Phi value*** |
| **LAT** | Intercept | 79.76 | 203.30 | <0.001 | s(day) | 1.99 | 2033.00 | <0.001 | 0.99 |
| **LON** | Intercept | 4.39 | 0.67 | 0.50 | s(day) | 1.99 | 356.50 | <0.001 | 0.99 |
| **DIST** | Intercept | 0.04 | 0.34 | 0.74 | s(day) | 1.98 | 27.17 | <0.001 | 0.80 |
| **Environmental metrics** | | | | | | | | |  |
|  |  | ***estimate*** | ***t-value*** | ***p***-value |  | edf | F | ***p-value*** | ***Phi value*** |
| ***DEP*** | Intercept | 6.36 | 24.82 | <0.001 | s(day) | 4.94 | 92.48 | <0.001 | 0.99 |
| ***ICE*** | Intercept | 3.06 | 17.30 | <0.001 | s(day) | 4.75 | 27.43 | <0.001 | 0.96 |
| ***ICE EDGE*** | Intercept | 35.58 | 3.85 | <0.001 | s(day) | 4.94 | 93.69 | <0.001 | 0.99 |
| ***SST*** | Intercept | 0.39 | 2.58 | <0.01 | s(day) | 4.39 | 55.22 | <0.001 | 0.98 |
| ***COAST*** | Intercept | 129.64 | 5.61 | <0.001 | s(day) | 4.99 | 1558.00 | <0.001 | 0.99 |

**Table S4.** GAMM model output summaries investigating TSA as a function of the environmental conditions within seasons. The estimates, *t-values* and *p-values* are shown for the linear predictor variables and the estimated degrees of freedom (edf), F and *p-values* are shown for the predictor variables included in the smooth function in each model. The level of temporal autocorrelation (phi value) is 0.28.

|  | ***Estimate*** | ***t value*** | ***p-value*** |
| --- | --- | --- | --- |
| Intercept | 1.06 | 9.36 | <0.001 |
| Spring | -0.24 | -0.54 | 0.59 |
| Summer | 0.04 | 0.44 | 0.66 |
| Winter | 0.09 | 0.60 | 0.55 |
|  | ***edf*** | ***F*** | ***p-value*** |
| s(DEP):Autumn | 2.25 | 7.41 | 0.001 |
| s(DEP):Spring | 1 | 0.91 | 0.34 |
| s(DEP):Summer | 1 | 5.76 | 0.02 |
| s(DEP):Winter | 1.89 | 10.78 | <0.001 |
| s(COAST):Autumn | 2.24 | 6.62 | <0.001 |
| s(COAST):Spring | 1 | 0.26 | 0.61 |
| s(COAST):Summer | 1 | 3.25 | 0.07 |
| s(COAST):Winter | 2.48 | 3.15 | 0.02 |
| s(ICE EDGE):Autumn | 1 | 0.08 | 0.78 |
| s(ICE EDGE):Spring | 2.10 | 2.42 | 0.06 |
| s(ICE EDGE):Summer | 2.27 | 1.08 | 0.20 |
| s(ICE EDGE):Winter | 2.00 | 3.75 | 0.02 |
| s(SST):Autumn | 1.89 | 4.92 | 0.03 |
| s(SST):Spring | 1 | 0.66 | 0.42 |
| s(SST):Summer | 2.65 | 4.56 | 0.01 |
| s(SST):Winter | 1 | 4.41 | 0.04 |
